# Prognostic heterogeneity in ASXL1-mutated AML and refinement by an immunophenotype-based score

**DOI:** 10.3389/fonc.2026.1780304

**Published:** 2026-05-19

**Authors:** Yaming Zhang, Min Zhang, Yali Huang, Junzhao Wan, Ping Liu, Ping Wang, Zhen Zhou, Fangya Hu, Jianta Wang, Dan Ma, Qian Kang

**Affiliations:** 1 Department of Hematology, Key Laboratory of Hematological Malignancies at Affiliated Hospital of Guizhou Medical University, Guiyang, China; 2 State Key Laboratory of Discovery and Utilization of Functional Components in Traditional Chinese Medicine, School of Pharmacy, Guizhou Provincial Engineering Technology Research Center for Chemical Drug R&D, Guizhou Medical University, Guiyang, China; 3 Department of Pharmacy, The Fourth People’s Hospital of Guiyang, Guiyang, China; 4 Natural Products Research Center of Guizhou Province, Guiyang, China

**Keywords:** acute myeloid leukemia (AML), ASXL1, immuScore, mutation, risk

## Abstract

**Introduction:**

*ASXL1* is a frequently mutated gene in acute myeloid leukemia (AML) and is clinically recognized as an adverse prognostic marker, yet its clinical impact remains markedly heterogeneous.

**Methods:**

Utilizing the BeatAML cohort, we integrated genomic, transcriptomic, immunophenotypic, and drug-sensitivity data to delineate this variability.

**Results:**

Although *ASXL1* mutations remained an independent adverse factor in multivariable analyses, survival outcomes did not differ significantly between *ASXL1*-mutated patients and those lacking other high-risk lesions after excluding co-occurring adverse mutations, suggesting that *ASXL1* status alone does not uniformly dictate prognosis. By stratifying *ASXL1*-mutated patients based on treatment response, we identified two biologically distinct subsets-complete remission (CR) versus relapsed/refractory (R/R)-characterized by divergent clinical outcomes. Comparative analyses revealed distinct transcriptional profiles, including upregulated MAP3K15 expression in *ASXL1*-mutated cases, alongside consistent immunophenotypic downregulation of CD11b, CD123, and HLA-DR in R/R patients. Leveraging these routinely measured markers, we developed a three-parameter ImmuScore that robustly stratified prognosis in both the BeatAML cohort and an independent clinical validation set. Importantly, the ImmuScore captured functional drug-response patterns: patients with low scores displayed significantly higher Z-scores-indicating reduced sensitivity across multiple chemotherapeutic and targeted agents.

**Discussion:**

These findings link immunophenotypic signatures to therapeutic vulnerability, providing a mechanistic explanation for treatment failure in a subset of *ASXL1*-mutated AML. To address potential small-sample bias, Firth penalized regression was applied. Collectively, this study demonstrates that *ASXL1*-mutated AML is not a monolithic high-risk entity. The ImmuScore provides a biologically informed and clinically feasible tool to identify truly high-risk *ASXL1*-mutated patients, potentially guiding personalized therapeutic strategies.

## Introduction

Acute myeloid leukemia (AML) is a genetically heterogeneous hematologic malignancy characterized by markedly variable clinical outcomes (1–5). Despite the integration of cytogenetic and molecular markers into modern stratification frameworks, such as ELN-2017 and ELN-2022, recent evidence suggests these models fail to fully encapsulate the biological diversity governing treatment response and relapse (6–10). This challenge is particularly pronounced in older patients and those harboring myelodysplasia-related mutations, a group enriched for alterations in epigenetic regulators and chromatin modifiers that drive clonal evolution, where therapeutic resistance remains a formidable clinical hurdle (11–15).

Additional sex combs like 1 (*ASXL1*) is among the most frequently mutated genes in AML and is consistently linked to an adverse prognosis (16–20). Consequently, *ASXL1* mutations are categorized within the ELN-2022 adverse-risk group (21, 22). However, emerging evidence indicates that the prognostic impact of *ASXL1* is highly context-dependent rather than uniformly deleterious. Several studies have demonstrated that *ASXL1* mutations predict inferior survival primarily in the presence of cooperating mutations-such as *RUNX1*, *NRAS*, or *TP53*-or within specific clinical contexts, including secondary AML or MDS-related AML (23–25). In alternative cohorts, survival disparities between *ASXL1*-mutated and mutation-negative patients are markedly attenuated (26, 27). These observations underscore the substantial heterogeneity within the *ASXL1*-mutated population and suggest that additional biological modifiers, beyond the mutation itself, are requisite for precise risk stratification (28–30).

Immunophenotypic markers assessed by multiparameter flow cytometry-including CD11b, CD123, and HLA-DR-are routinely evaluated at diagnosis and have been associated with treatment response, leukemic differentiation, and stemness biology in AML (31–36). Importantly, these markers reflect functional cellular states that cannot be fully captured by genomic data alone, offering a complementary layer of biological insight. However, their prognostic significance has seldom been evaluated within genetically defined subgroups, such as *ASXL1*-mutated AML. Whether integrating immunophenotypic features with genetic alterations could refine risk stratification and identify truly high-risk patients remains elusive and has not been systematically investigated (37, 38).

In the present study, utilizing the BeatAML cohort, we confirmed that *ASXL1* mutation independently predicted inferior survival; however, we also observed that overall survival did not differ significantly when *ASXL1*-mutated patients were compared with those lacking any driver mutations (20, 27, 39). This inconsistency reinforced the notion that *ASXL1* mutation alone is insufficient to account for clinical heterogeneity. We therefore hypothesized that immunophenotypic modifiers may drive this variability. By integrating the expression of CD11b, CD123, and HLA-DR-three markers routinely reported in clinical AML flow cytometry-we developed a composite immunophenotypic score (ImmuScore) to refine risk stratification within *ASXL1*-mutated AML. The robustness of ImmuScore was subsequently validated in an independent patient cohort (40).

A detailed overview of the study design and analytical workflow is provided in **Supplementary Figure 3**, which outlines our systematic integration of genomic, immunophenotypic, transcriptomic, and drug-response analyses throughout the study.

## Materials and methods

### Data acquisition

Patient-level clinical and genomic information for the BeatAML cohort were retrieved from the BloodPAC Data Commons (https://biodev.github.io/BeatAML2/). The dataset encompasses demographic characteristics, mutational annotations (derived from a standardized sequencing panel), cytogenetics, *ex vivo* drug sensitivity profiles, flow cytometry-derived immunophenotypic data, and overall survival outcomes. To ensure comprehensive genomic characterization, these multi-omic layers were integrated to construct an OncoPrint summarizing the genomic landscape, cytogenetic risk, clinical features, and ImmuScore distribution. All data were fully de-identified prior to acquisition. The use of BeatAML data complied with all ethical provisions and data-use agreements established by the original investigators.

### Statistical analysis

#### Multivariable survival analysis

The association between clinical variables and overall survival (OS) was evaluated using multivariable Cox proportional hazards regression models. OS was defined as the interval from the date of diagnosis to death from any cause; patients who remained alive were censored at the date of their last follow-up. Covariates included in the initial models were selected based on clinical relevance and findings from preceding univariable analyses. Independent prognostic factors were identified and reported as hazard ratios (HRs) with corresponding 95% confidence intervals (CIs). All statistical analyses were performed using R software. To ensure consistency across endpoints, Cox models were uniformly applied to OS unless otherwise specified.

#### Variable screening using firth regression

Univariable screening was performed to identify variables associated with OS among *ASXL1*-mutated patients, with a specific focus on features differentiating complete remission (CR) from relapsed/refractory (R/R) disease. Given the relatively small sample size of the *ASXL1*-mutated cohort and the high-dimensional nature of the dataset-encompassing biochemical, hematological, and genetic variables-we employed Firth’s penalized-likelihood Cox regression. This method was specifically selected to mitigate bias in maximum likelihood estimation (MLE), thereby yielding more precise and stable hazard ratios (HRs). Statistically significant variables identified through this screening process were prioritized for subsequent multivariable modeling. Given that *ASXL1* mutations occur in a minority of AML cases (approximately 50 patients per cohort in this study), Firth penalization was instrumental in addressing small-sample bias and potential issues of “separation” in the regression models. For analyses involving binary treatment response (CR vs. R/R) as the primary endpoint, Firth-penalized logistic regression was additionally utilized to ensure robust parameter estimation under sparse-event conditions.

#### Survival analysis visualization

Overall survival (OS) was defined as the interval from the date of diagnosis to death from any cause; data from patients who were alive at the time of last follow-up were censored. Survival probabilities over time were estimated and visualized using Kaplan-Meier curves, generated via the survival and survminer packages in R. Specifically, survival functions were fitted using the survfit function, and publication-quality graphics were rendered using ggsurvplot. The resulting plots include risk tables and *P*-values derived from the log-rank test, which was employed to assess the statistical significance of survival disparities between cohorts. Additionally, forest plots were utilized to summarize HRs and their corresponding 95% CIs, enabling a streamlined visual comparison of independent prognostic factors.

#### Immunophenotypic profiling and differential expression analysis

Flow cytometry data from the BeatAML cohort were processed to generate a quantitative immunophenotype matrix. To facilitate computational analysis, the categorical expression levels of each antibody were converted into numerical scores based on the following pre-defined criteria: a score of 1.0 was assigned to “bright” positive expression; 0.5 to a general positive pattern (“part”); and 0.2 to “dim” or partial-dim (“partdim”) expression; 0 to negative expression. This transformation resulted in a structured patient-by-antibody matrix, enabling rigorous comparative downstream analyses.

To identify markers with significant differential expression between the defined patient subgroups, we utilized the limma package in R. This approach employs a linear modeling framework moderated by empirical Bayes statistics, ensuring robustness in high-dimensional data settings. This analysis yielded a list of statistically significant differentially expressed antibodies, which were subsequently visualized using clustered heatmaps annotated by clinical group membership. While several markers, such as CD58 and CD71, exhibited significant variance between CR and R/R patients, they are not universally included in routine diagnostic AML flow cytometry panels. Conversely, CD11b, CD123, and HLA-DR are systematically reported in standard clinical practice. Consequently, these three markers were selected for model construction to maximize cross-center reproducibility and clinical translational feasibility.

### ImmuScore calculation and group stratification

To establish a robust and clinically applicable immunophenotypic signature, we developed a simplified scoring system termed the “ImmuScore”. For each patient, the ImmuScore was derived by aggregating the quantitative flow cytometry scores-previously assigned based on expression intensity-for three key markers: CD11b, CD123, and HLA-DR.

To define clinically meaningful risk groups, patients within each cohort were dichotomized based on their respective ImmuScore distributions. Specifically, the median ImmuScore was determined independently for the BeatAML discovery dataset and the institutional validation cohort. Patients with an ImmuScore greater than or equal to the cohort-specific median were classified into the “High Score” group, while those with a score below the median were assigned to the “Low Score” group. This median-based stratification approach was intentionally selected to circumvent potential platform-dependent scaling discrepancies and to ensure the consistent, reproducible application of the scoring system across independent cohorts and diverse clinical settings.

### Gene expression and differential analysis

Gene expression data for the BeatAML cohort, quantified as Transcripts Per Million (TPM), were log_2_-transformed (following the addition of a pseudo-count of 1) to meet the distributional assumptions required for linear modeling. Differential expression analysis between comparison groups was performed using the limma (Linear Models for Microarray Data) package in R. This process involved fitting linear models for each gene, followed by empirical Bayes moderation to shrink estimated sample variances toward a common value, thereby enhancing the statistical power and robustness of the analysis. The significance of expression changes was evaluated using moderated t-statistics. Genes were identified as significantly differentially expressed based on a dual threshold: an absolute log_2_ fold-change (|log_2_FC|) > 1 and an adjusted *P*-value < 0.05. Where applicable, significantly deregulated genes underwent functional enrichment analysis to delineate the mechanistic links between transcriptomic alterations, immunophenotypic signatures, and drug-sensitivity patterns.

### Identification of survival-associated genes via lasso cox regression

To identify a parsimonious set of genes with the highest predictive value for patient survival, we subjected the differentially expressed genes (DEGs) to a Least Absolute Shrinkage and Selection Operator (Lasso) penalized Cox proportional hazards regression analysis. The Lasso method, implemented using the glmnet R package, applies an *L*_1_-penalty to the regression coefficients, effectively shrinking the coefficients of non-informative or redundant variables to zero.

This approach facilitates robust variable selection by mitigating overfitting and addressing potential multicollinearity within the high-dimensional transcriptomic data. The optimal penalty parameter (λ) was determined through 10-fold cross-validation, specifically selecting the value that minimized the partial likelihood deviance (λ_min_) to ensure maximum predictive accuracy. Genes retained in the final model with non-zero coefficients were defined as key survival-associated signatures. Model discrimination was evaluated using time-dependent Receiver Operating Characteristic (ROC) curves, and internal validation was performed via bootstrap resampling to adjust for optimism in performance estimates.

### Ethics statement

The studies involving human participants were reviewed and approved by the Ethics Committee of the Affiliated Hospital of Guizhou Medical University (Approval No. 2020-081). This research was conducted in strict accordance with local legislation and institutional requirements. In alignment with Chinese national regulations, since the clinical materials used in this study were originally collected by the Affiliated Hospital of Guizhou Medical University for routine diagnostic and therapeutic purposes and were analyzed in an anonymized format for research, the requirement for written informed consent was waived by the ethics committee.

### Clinical data collection and validation cohort

To evaluate the robustness and clinical utility of the ImmuScore, we established an independent validation cohort comprising 49 patients with ASXL1-mutated acute myeloid leukemia (AML) treated at the Affiliated Hospital of Guizhou Medical University between 2020 and 2025. This cohort included both newly diagnosed and relapsed cases. Comprehensive clinical records were systematically curated, encompassing longitudinal hematological and biochemical profiles, cytogenetic and molecular genetic data, and multiparameter flow cytometry-derived immunophenotypes. Furthermore, detailed therapeutic histories and overall survival (OS) outcomes were integrated to facilitate a rigorous assessment of the ImmuScore’s performance in a real-world clinical setting.

### Sanger sequencing for *ASXL1* mutation detection

Genomic DNA was isolated from bone marrow mononuclear cells using a commercial extraction kit, adhering strictly to the manufacturer’s protocol. DNA concentration and purity were quantified via spectrophotometry. Given that the majority of pathogenic truncating mutations in *ASXL1* cluster within the 3’ end of the gene, Exon 12 was amplified by polymerase chain reaction (PCR) using sequence-specific primers flanking the mutational hotspot region. PCR was conducted under optimized cycling conditions, and the integrity of the resulting amplicons was confirmed by agarose gel electrophoresis. Purified PCR products underwent bidirectional Sanger sequencing on an ABI 3730xl DNA Analyzer (Applied Biosystems). Sequencing chromatograms were processed and aligned to the *ASXL1* reference transcript (NM_015338). Mutations were identified through the systematic inspection of electropherograms and, where necessary, validated by independent replicate sequencing. Only variants exhibiting clear, reproducible signal patterns were considered for downstream analysis. All identified *ASXL1* alterations were annotated in accordance with the Human Genome Variation Society (HGVS) nomenclature.

### Flow cytometry analysis of bone marrow malignant populations

Bone marrow aspirates were collected in EDTA-anticoagulated tubes and processed within 4 hours of acquisition to ensure optimal cell viability. Samples underwent erythrocyte lysis using an ammonium chloride-based lysis buffer (BD Biosciences) for 5–7 minutes at room temperature, followed by two washes in phosphate-buffered saline (PBS) supplemented with 2% fetal bovine serum (FBS). Cell suspensions were filtered through a 40 μm nylon mesh to remove aggregates and adjusted to a final concentration of 1-2 × 10^6^ cells per test.

For immunophenotypic profiling of malignant blasts, cells were incubated with a pre-titrated multicolor antibody panel for 20 minutes at 4°C in the dark. The core panel used to define leukemia-associated immunophenotypes (LAIPs) comprised CD45, CD34, CD117, HLA-DR, CD33, CD13, CD38, and CD123. Where indicated, additional markers-including CD7, CD56, CD19, and CD11b-were incorporated to characterize aberrant differentiation or lineage infidelity. Following surface staining, cells were washed and resuspended in 300 µL of PBS/2% FBS for acquisition.

Data acquisition was performed on a BD FACSLyric system (BD Biosciences) using standardized photomultiplier tube (PMT) settings and daily CS&T (Cytometer Setup and Tracking) calibration to ensure longitudinal consistency. A minimum of 100, 000 live singlet events were recorded per sample. The malignant blast population was identified using a canonical CD45 versus side scatter (SSC) gating strategy, followed by sequential refinement based on immature markers (CD34, CD117) and lineage-specific antigen expression. Data were analyzed using BD FACSuite software. The malignant cell burden was quantified as the percentage of LAIP-positive blasts relative to total viable nucleated cells.

### Primer design and *in silico* specificity validation

The coding sequence (CDS) of the target gene was retrieved from the NCBI Nucleotide database (GenBank). Specific primer pairs flanking the regions of interest were designed using the primer design module in SnapGene software. Design parameters were optimized with an amplicon length of 80–200 base pairs, a primer melting temperature (T_m_) of 58-62°C, and a GC content of 40-60%. To ensure high amplification efficiency and suppress secondary structures, primers were screened for potential hairpins and self-dimers. The specificity of the candidate primer sequences was subsequently validated in silico using the NCBI BLASTN tool against the RefSeq mRNA database. This step was performed to confirm unique binding to the intended transcript, thereby minimizing non-specific amplification or cross-reactivity with homologous sequences or pseudogenes. Only primer pairs exhibiting high sequence complementarity and a significant E-value were selected for downstream experimental validation.

### RNA isolation and quantitative real-time PCR

Bone marrow aspirates were collected from AML patients with clinically confirmed *ASXL1* mutations. Total RNA was isolated from these samples using a commercial kit according to the manufacturer’s instructions. RNA integrity and concentration were verified prior to reverse transcription. Complementary DNA (cDNA) was synthesized from a fixed amount of total RNA using a reverse transcriptase kit. Gene expression levels were then quantified by real-time fluorescence quantitative PCR (RT-qPCR) on a designated platform. The reaction mixture contained cDNA template, gene-specific forward and reverse primers, and a SYBR Green master mix. The relative expression of target genes was calculated using the comparative 2^(-ΔΔCt) method, with *GAPDH* (or another appropriate housekeeping gene) used as an endogenous control for normalization. For *MAP3K15*, relative expression levels were compared between *ASXL1*-mutated and wild-type samples to validate bioinformatic findings from the BeatAML RNA-Seq analysis. The primer sequences utilized were as follows: *GAPDH*: Forward 5’- AGATCCCTCCAAAATCAAGTGG-3’; Reverse 5’-GGCAGAGATGATGACCCTTTT-3’; *MAP3K15*: Forward 5’-CGGCTGAACAGTTTGTTGGG-3’; Reverse 5’- TGACGCTGAAGAACTGACCC-3’.

### Immune-cell deconvolution and microenvironment analysis

Immune cell fractions in the tumor microenvironment were quantified using CIBERSORT. Gene expression data were normalized to non-log linear space and deconvoluted using the LM22 signature matrix, which distinguishes 22 immune cell subsets. CIBERSORT was run with 1, 000 permutations, and samples with deconvolution *P* < 0.05 were considered reliable and retained for downstream analysis. Differences in immune cell composition between groups were compared using the Wilcoxon rank-sum test, with statistical significance set at *P* < 0.05. Results were visualized using boxplots.

## Results

### Prognostic significance of clinical and molecular variables in the beataml cohort

**Figure 1:**
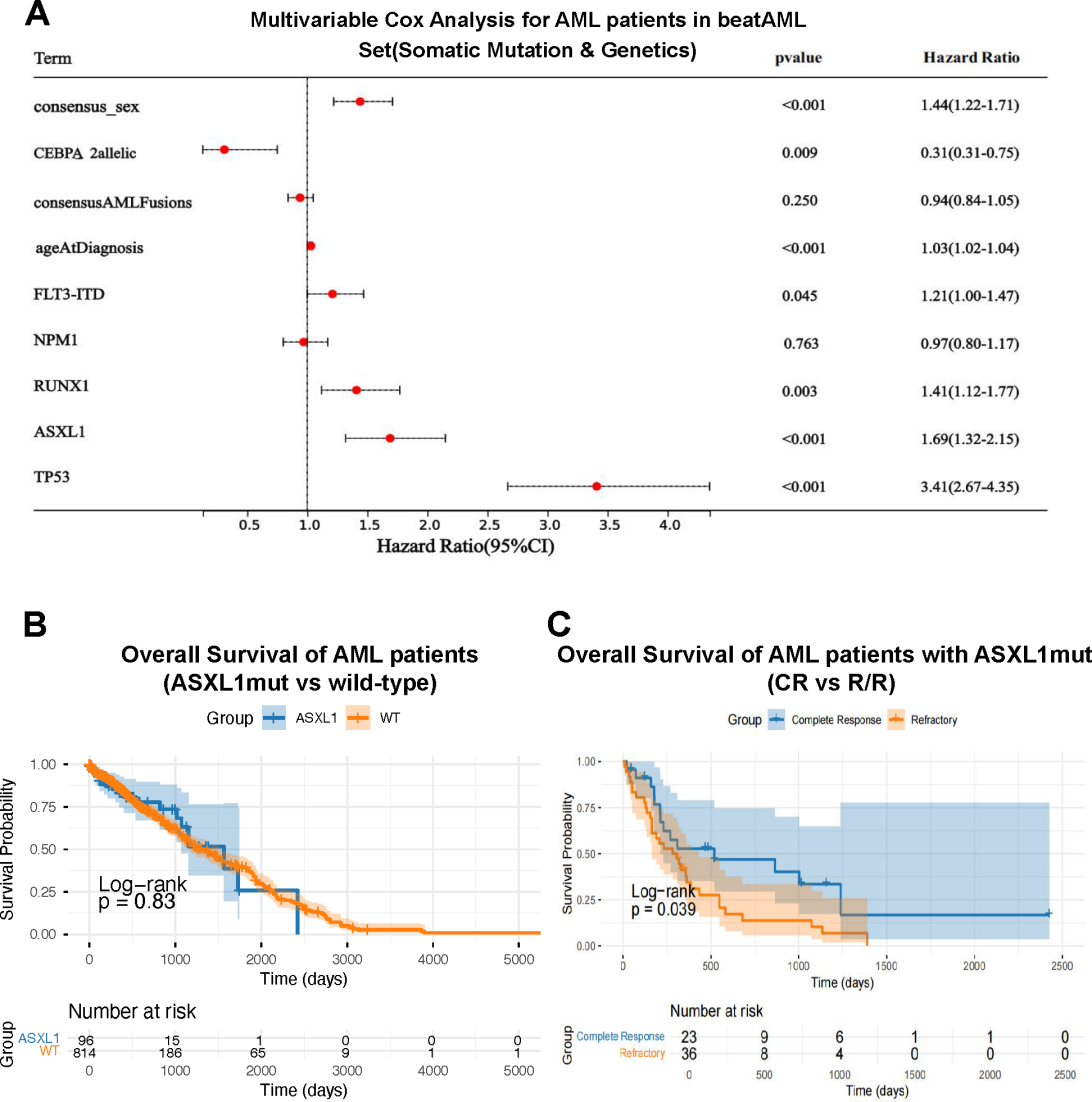
Prognostic significance of *ASXL1* mutations in AML (BeatAML cohort). **(A)** Multivariable Cox regression analysis of recurrent somatic mutations and cytogenetic abnormalities in AML patients from the BeatAML cohort. Hazard ratios (HR) with 95% confidence intervals are shown. **(B)** Kaplan-Meier survival curves comparing overall survival (OS) between *ASXL1*-mutated patients and those without detectable driver mutations. Log-rank *P-*values and numbers at risk are indicated. **(C)** Kaplan-Meier OS curves of *ASXL1*-mutated patients stratified by treatment response [complete remission (CR) vs refractory/relapsed (R/R)].

Somatic mutations and chromosomal rearrangements are primary determinants of clinical outcomes in acute myeloid leukemia (AML). Utilizing the comprehensive BeatAML dataset (n > 500), we first performed multivariable Cox regression analysis to reassess the prognostic contribution of common driver alterations (**Supplementary Figure 1A**; **Figure 1A**). Consistent with established literature and the ELN 2017/2022 stratification frameworks, mutations in *TP53*, *ASXL1*, and *RUNX1* emerged as independent adverse prognostic factors, whereas biallelic CEBPA mutations were associated with favorable survival. Notably, ASXL1 exhibited the second-highest hazard ratio (HR)-surpassed only by *TP53*-underscoring its clinical significance and establishing it as a critical candidate for further investigation.

Despite this well-recognized adverse association, emerging evidence suggests that the prognostic impact of *ASXL1* may be context-dependent rather than monolithic. To delineate its independent contribution, we compared *ASXL1*-mutated patients with those lacking other established high-risk lesions as defined in **Figure 1A**. Intriguingly, overall survival (OS) did not differ significantly between these two groups (log-rank *P* = 0.83; **Figure 1B**). This unexpected finding contrasts with the uniformly adverse classification of *ASXL1* in current guidelines and suggests that *ASXL1*-mutated AML encompasses biologically heterogeneous subsets whose clinical risk is not fully captured by mutational status alone.

To further explore this heterogeneity, we stratified *ASXL1*-mutated patients based on initial treatment response. A striking divergence in clinical trajectories was observed: patients achieving complete remission (CR) exhibited markedly superior survival compared to those with relapsed/refractory (R/R) disease (log-rank *P* = 0.039; **Figure 1C**). Crucially, this survival disparity persisted despite an identical *ASXL1* mutational profile, indicating that *ASXL1* status alone is insufficient to account for the substantial clinical variability observed.

Collectively, these analyses yield two pivotal insights. First, the prognostic effect of *ASXL1* mutations is strongly modulated by additional biological factors-such as co-occurring mutations or individual treatment response-that dictate its ultimate clinical impact. Second, reliance on mutational status alone may obscure clinically meaningful subgroups within *ASXL1*-mutated AML. These observations motivated our subsequent efforts to identify immunophenotypic and functional markers capable of explaining the divergent trajectories between CR and R/R patients, providing the foundation for a refined risk-stratification framework.

### Functional drug-sensitivity profiling of *ASXL1*-mutated AML subsets

After identifying the immunophenotypic and transcriptomic features associated with prognosis, we sought to determine whether functional variations in pharmacological response further contribute to the clinical heterogeneity between CR and R/R subgroups within *ASXL1*-mutated AML. Evaluating drug sensitivity provides a critical translational bridge, linking identified biomarkers to individualized therapeutic strategies for this genetically defined population.

**Figure 2:**
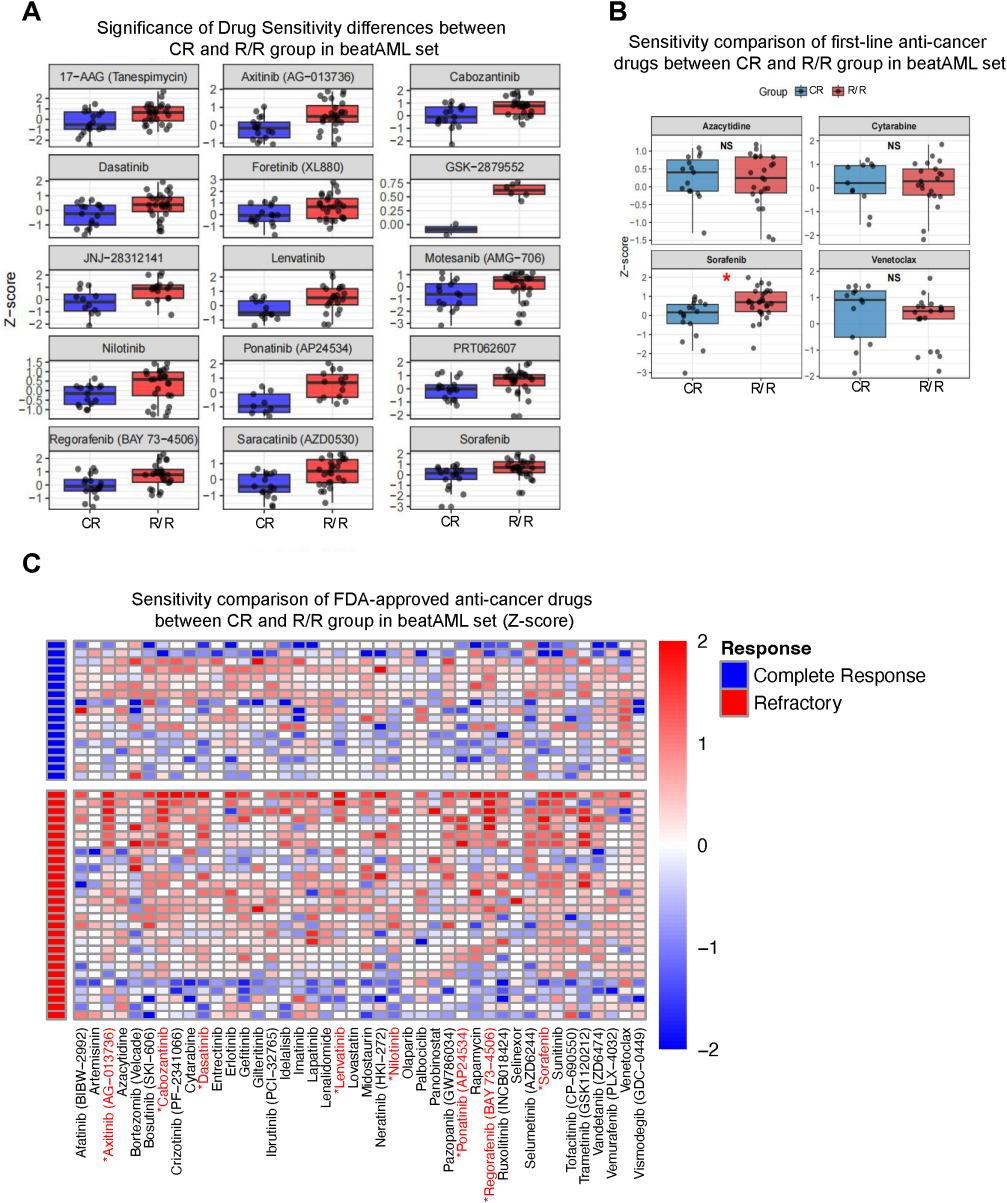
Comparative drug sensitivity profiling of CR and R/R subgroups. **(A)** Boxplots showing the distribution of drug sensitivity (Z-score) for significantly different compounds between complete response (CR) and refractory/relapsed (R/R) groups. Several tyrosine kinase inhibitors (e.g., Axitinib, Foretinib, Lenvatinib, Ponatinib, Regorafenib, and Sorafenib) displayed significantly higher sensitivity in the CR group compared to the R/R group. **(B)** Sensitivity comparison of first-line anti-leukemic drugs (Azacitidine, Cytarabine, Sorafenib, and Venetoclax) between CR and R/R patients. Among them, Sorafenib showed significantly increased sensitivity in CR patients, while no significant differences were observed for the others. **(C)** Heatmap illustrating the sensitivity profiles (Z-scores) of FDA-approved anti-cancer drugs across CR and R/R groups. The analysis highlights broad patterns of reduced sensitivity in the R/R group, supporting the notion of intrinsic drug resistance in these patients.

Drug response data from the BeatAML dataset were normalized using Z-scores to facilitate cross-compound comparisons. When analyzing the comprehensive drug library (**Figure 2A**), the CR subgroup exhibited significantly higher sensitivity to multiple multi-target tyrosine kinase inhibitors (TKIs), including axitinib, foretinib, lenvatinib, ponatinib, and regorafenib. This consistent trend across structurally diverse TKIs suggests that CR patients may harbor specific signaling dependencies-potentially linked to the *ASXL1*-driven epigenetic landscape-that remain pharmacologically vulnerable, whereas the R/R subgroup displays a more intrinsically resistant phenotype.

We further scrutinized frontline clinical agents utilized in AML therapy (**Figure 2B**). Notably, sorafenib demonstrated markedly higher potency in the CR subgroup compared to the R/R group. Conversely, no significant disparities were observed for standard-of-care agents such as azacitidine, cytarabine, or venetoclax. These findings imply that while certain targeted therapies may preferentially benefit patients in the CR-trajectory, standard chemotherapeutics fail to adequately address the biological drivers of resistance in the R/R subset. This reinforces the necessity of incorporating biological modifiers-such as the immunophenotype and transcriptomic signatures identified earlier-into therapeutic decision-making.

Further analysis of a broader panel of FDA-approved anti-cancer agents (**Figure 2C**) revealed that the majority did not exhibit differential sensitivity between the two subgroups. This observation aligns with the characterization of the R/R subgroup as a functionally refractory population in which conventional mechanisms of drug susceptibility are largely attenuated.

Collectively, these results demonstrate that functional drug profiling adds a critical dimension to risk stratification in *ASXL1*-mutated AML. The convergence of distinct immunophenotypic signatures, transcriptomic profiles, and drug-response patterns highlights a coherent biological framework in which R/R patients represent a more resistant and clinically unfavorable subclass. Importantly, none of the tested agents demonstrated preferential ex vivo sensitivity in the R/R subgroup, indicating that novel treatment strategies-potentially informed by the biomarkers identified in the subsequent analyses-or rational drug combinations may be necessary to improve outcomes for this high-risk population.

### Identification of biological determinants distinguishing CR and R/R trajectories in *ASXL1*-mutated AML patients

To elucidate the biological underpinnings of the prognostic heterogeneity observed in *ASXL1*-mutated AML, we performed a comprehensive comparative analysis of clinical characteristics, co-mutation landscapes, and immunophenotypic profiles between patients achieving complete remission (CR) and those with relapsed/refractory (R/R) disease. This approach aimed to identify modifiers that cooperate with *ASXL1* mutations to drive divergent clinical outcomes.

**Figure 3:**
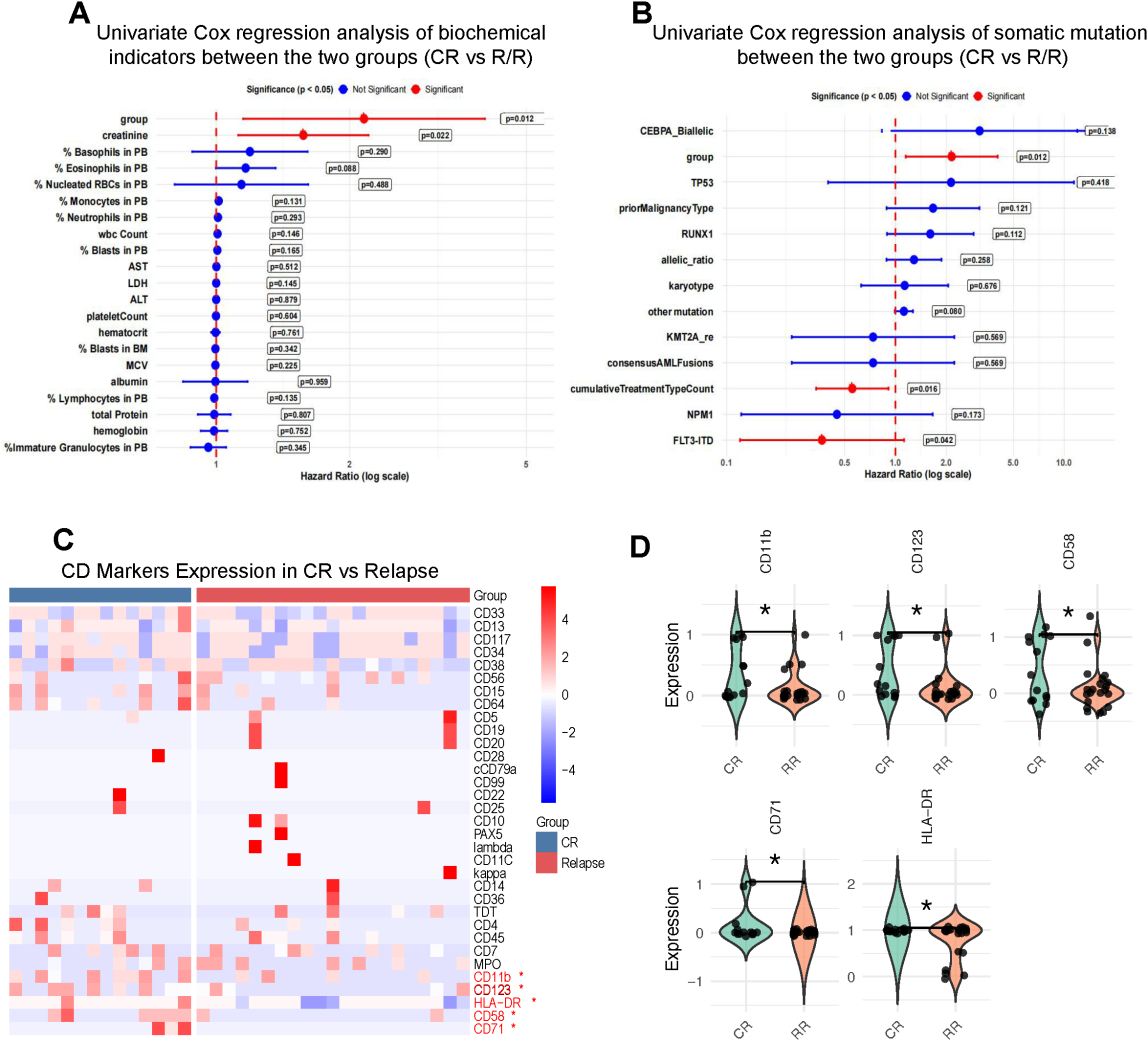
Clinical, genetic, and immunophenotypic determinants of CR and R/R trajectories. **(A)** Univariate Cox regression analysis of biochemical indicators between patients achieving complete remission (CR) and those with relapsed/refractory (R/R) disease. Hazard ratios with 95% confidence intervals are shown; serum creatinine was significantly associated with prognosis (*P* = 0.022). **(B)** Univariate Cox regression analysis of recurrent somatic mutations and clinical variables between CR and R/R groups. Prior malignancy history, cumulative treatment type count, and FLT3-ITD mutation were significantly correlated with adverse prognosis. **(C)** Heatmap of antigen expression profiles detected by multiparameter flow cytometry (FCM) in CR versus R/R patients. Several markers, including CD11b, CD123, and HLA-DR, displayed differential expression between the two groups. **(D)** Violin plots illustrating differential expression of representative antigens (CD11b, CD123, CD71, CD58 and HLA-DR) between CR and R/R patients, with lower expression observed in the R/R group. Each dot represents one patient. Boxplots indicate median and interquartile range. P values were calculated using the Wilcoxon rank-sum test with exact = FALSE. * Representing p <0.05.

We first evaluated conventional clinical and biochemical variables (**Figure 3A**). Univariate Cox regression identified only serum creatinine levels as significantly associated with prognosis (*P* = 0.022), suggesting that impaired renal function may adversely impact patient survival. Notably, other established hematologic and biochemical indicators-including white blood cell count, platelet count, LDH levels, and baseline bone marrow blast percentage (ranging from 2% to 95%)-showed no significant correlation with survival outcomes. These results underscore that traditional laboratory metrics are insufficient to account for the pronounced CR/RR divergence within the *ASXL1*-mutated population.

We next examined the impact of genetic co-alterations and clinical history (**Figure 3B**). Factors such as the cumulative number of prior treatment modalities received (*P* = 0.016) and the presence of FLT3-ITD mutations (*P* = 0.042) were associated with inferior prognosis. However, several recurrent driver mutations-including *NPM1*, *RUNX1*, and *TP53*-failed to significantly differentiate the CR and R/R subgroups. Collectively, these findings suggest that while specific clinical histories and high-risk co-mutations modulate outcomes, they do not fully encapsulate the robust heterogeneity inherent in *ASXL1*-mutated AML, reinforcing the necessity for additional biological classifiers.

Given the limitations of standard variables, we leveraged multiparameter flow cytometry to explore immunophenotypic disparities (**Figures 3C, D**). This analysis revealed a consistent and significant separation between CR and R/R patients across five surface antigens: CD11b, CD58, CD123, CD71, and HLA-DR. Intriguingly, the R/R subgroup exhibited uniformly lower expression of these markers. As these antigens are critically involved in myeloid differentiation, immune-synapse formation, and therapeutic sensitivity, their coordinated downregulation may reflect a more primitive, immune-evasive, or stem-like leukemic phenotype in R/R patients.

In summary, this systematic comparison demonstrates that neither standard clinical metrics nor common co-mutations adequately explain the clinical heterogeneity within *ASXL1*-mutated AML. In contrast, immunophenotypic profiling provides a robust and biologically coherent distinction between CR and R/R subgroups. These insights provided the rationale for the development of the ImmuScore, a composite immunophenotypic scoring system designed to refine prognostic assessment for this genetically defined AML subgroup.

### Development and validation of a clinically translatable immuScore model

As immunophenotypic disparities provided the most robust and consistent separation between CR and R/R subsets in *ASXL1*-mutated AML, we sought to translate these biological insights into a practical prognostic tool. While five antigens (CD11b, CD58, CD123, CD71, and HLA-DR) were initially identified, only CD11b, CD123, and HLA-DR are systematically incorporated into standard diagnostic flow cytometry panels. To maximize clinical feasibility and ensure seamless integration into existing diagnostic workflows, we prioritized these three routinely assessed markers to construct a simplified composite scoring system, termed the ImmuScore.

**Figure 4:**
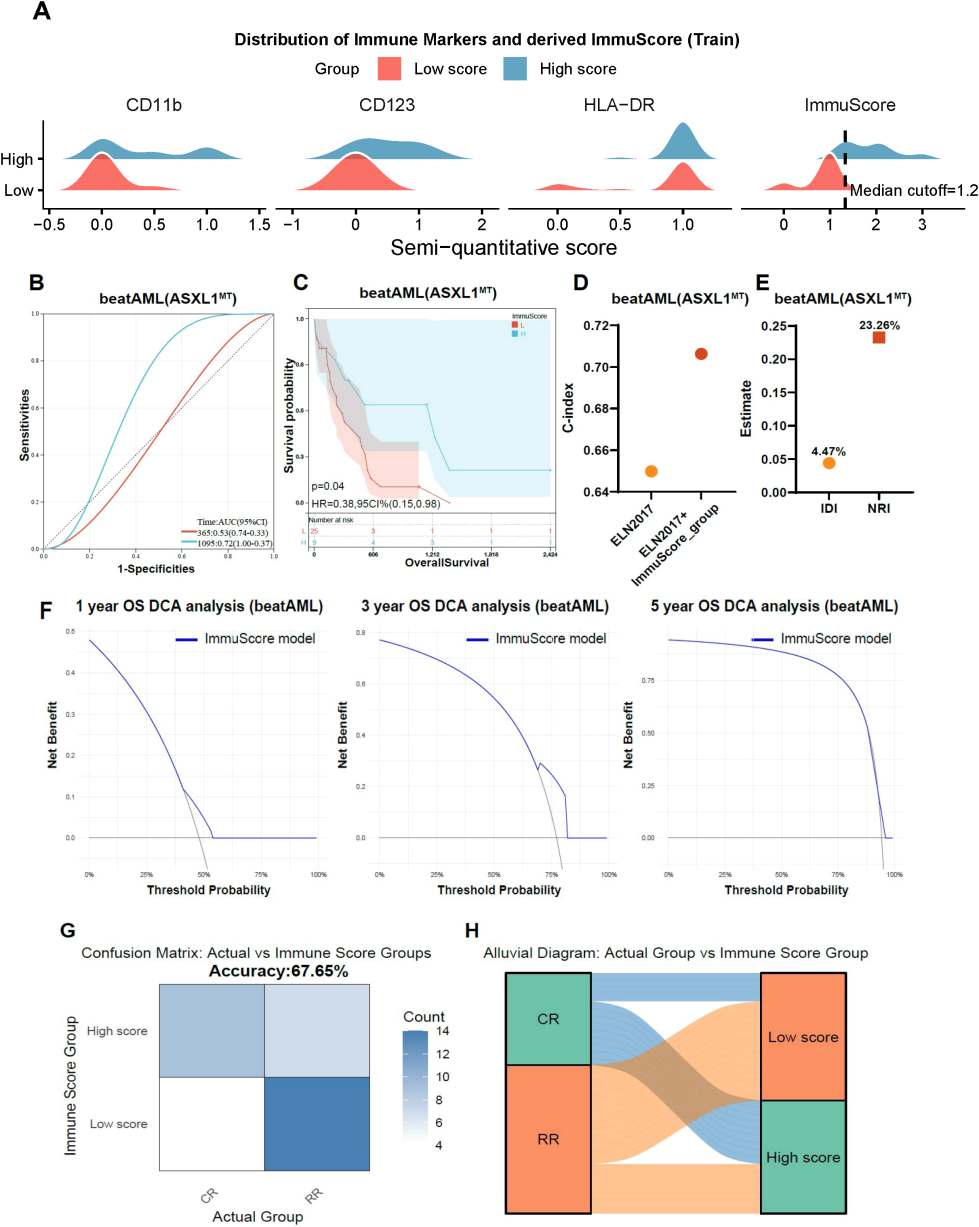
Predictive performance and clinical utility of the ImmuScore model. **(A)** Distribution of immune marker expression (CD11b, CD123, HLA-DR) stratified by ImmuScore-defined groups, Low-Score group showed reduced CD11b, CD123, and/or HLA-DR expression, whereas High-Score patients retained higher expression of these markers. **(B)** ROC curve analysis demonstrating the discriminatory performance of the ImmuScore for overall survival prediction in *ASXL1*-mutated patients. **(C)** Kaplan-Meier survival analysis indicating that patients with high ImmuScore had significantly longer overall survival compared with low-score patients (*P* = 0.04). **(D)** Concordance index (C-index) analysis comparing ImmuScore with ELN-2017 classification, showing comparable or slightly higher predictive performance relative to ELN-2017. **(E)** Integrated Discrimination Improvement (IDI) and Net Reclassification Improvement (NRI) analyses quantifying the incremental prognostic value of ImmuScore. **(F)** Decision curve analyses (DCA) for 1-, 3-, and 5-year overall survival, demonstrating clinical utility of the ImmuScore model across multiple time horizons. **(G)** Confusion matrix showing classification accuracy (67.65%) of the ImmuScore in distinguishing CR and R/R patients. **(H)** Alluvial diagram visualizing the concordance between clinical response categories (CR, R/R) and immune grouping defined by the ImmuScore.

Each antigen was semi-quantitatively scored based on expression intensity (strong = 1.0; intermediate = 0.5; weak = 0.2; negative = 0). In the training set of the BeatAML cohort, the calculated ImmuScore values showed a distributed pattern that allowed patient stratification using a cutoff value of 1.2, thereby separating cases into High-score and Low-score groups (**Figure 4A**). Performance benchmarking confirmed that this parsimonious three-marker model retained high predictive accuracy, performing comparably to the more complex five-marker version (**Supplementary Figures 2A, B**, **S4**). Time-dependent ROC analysis yielded an Area Under the Curve (AUC) of 0.72 for predicting 3-year overall survival (OS) (**Figure 4B**). Consistently, High-Score patients exhibited significantly superior survival compared to the Low-Score group (HR = 0.38, 95% CI: 0.15-0.98, *P* = 0.04; **Figure 4C**).

When benchmarked against the ELN-2017 classification, the ImmuScore achieved a concordance index (C-index) of approximately 0.70-comparable to and slightly exceeding ELN-2017 performance (**Figure 4D**). Integrated Discrimination Improvement (IDI) and Net Reclassification Index (NRI) analyses demonstrated a positive trend toward enhanced risk discrimination, although statistical significance was not reached (**Figure 4E**). Decision curve analysis (DCA) further confirmed a consistent net clinical benefit across a broad range of threshold probabilities for 1- and 3-year survival predictions (**Figure 4F**). To validate its link to clinical outcomes, the ImmuScore was compared against actual CR/RR status, yielding an overall classification accuracy of 67.65% (**Figure 4G**). An alluvial diagram (**Figure 4H**) further visualized the biological validation: CR cases predominantly clustered in the High-Score group, whereas R/R cases were concentrated in the Low-Score group.

Finally, to explore the microenvironmental landscape associated with these scores, immune-cell deconvolution analysis was performed. While most immune subsets remained comparable between groups, regulatory T cells (Tregs) were significantly depleted in R/R (Low-Score) patients (*P* < 0.001; **Supplementary Figure 2D**). This suggests a profound imbalance in immune regulation that may contribute to therapeutic failure in the Low-Score population.

### Transcriptomic profiling identifies *MAP3K15* as a complementary prognostic biomarker

Since the BeatAML cohort provides high-depth RNA-Seq data coupled with clinical outcomes, we investigated whether transcriptional alterations could further elucidate the prognostic heterogeneity observed in *ASXL1*-mutated AML. This analysis was conceptually pivotal, as gene-expression patterns may capture biological variations-such as intracellular signaling flux-that are complementary to, yet distinct from, the surface immunophenotypic signatures identified by flow cytometry.

**Figure 5:**
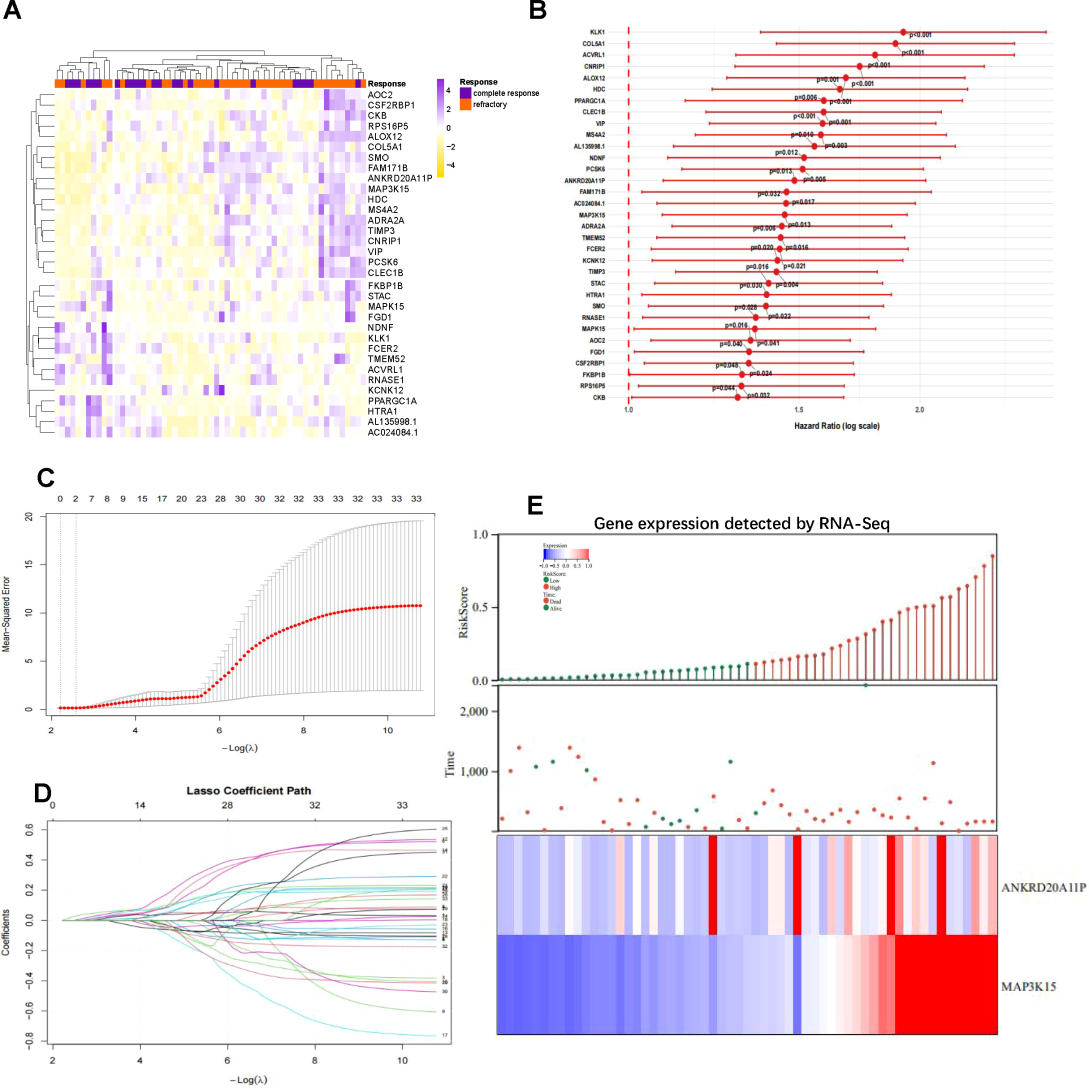
Transcriptomic signature identification and construction of the *MAP3K15*-based risk model. **(A)** Heatmap of differentially expressed genes between complete remission (CR) and relapsed/refractory (R/R) subgroups detected by RNA-Seq. **(B)** Univariate Cox regression analysis of gene expression associated with overall survival in *ASXL1*-mutated patients. Hazard ratios with 95% confidence intervals and p values are shown. **(C)** Ten-fold cross-validation for tuning parameter selection in the LASSO regression model. The red dots represent mean cross-validated error, and error bars indicate standard deviation. **(D)** LASSO coefficient profiles of prognostic genes as a function of the log(λ) value. Genes with non-zero coefficients at the optimal λ are retained in the model. **(E)** Final prognostic model constructed based on *ANKRD20A11P* and *MAP3K15* expression. Upper panel, risk score distribution of patients; middle panel, survival status; lower panel, heatmap of the two prognostic genes. Patients in the high-risk group exhibited significantly worse survival compared with the low-risk group.

Differential gene expression analysis revealed a distinct transcriptomic signature separating the CR and R/R subgroups (**Figure 5A**). Univariate Cox regression identified several genes significantly associated with overall survival (OS), reinforcing the potential of RNA-level alterations as independent prognostic indicators (**Figure 5B**). To address the high dimensionality and inherent redundancy of transcriptomic data, we employed LASSO Cox regression to isolate a parsimonious gene set with the highest independent predictive value. 10-fold cross-validation at the optimal penalty parameter (lambda) yielded a minimal set of informative genes with non-zero coefficients (**Figures 5C, D**). Two candidates-*ANKRD20A11P* and *MAP3K15*-emerged as the most robust predictors and were used to construct a concise gene-expression-based risk model (**Figure 5E**). Risk stratification based on this model demonstrated significantly inferior survival in the high-risk group, confirming that transcriptional features provide meaningful prognostic discrimination within the *ASXL1*-mutated population.

Given that *ANKRD20A11P* is a pseudogene with limited functional annotation, our subsequent mechanistic focus prioritized *MAP3K15*. *MAP3K15* encodes a stress-responsive MAP kinase with putative roles in leukemic cell survival and chemoresistance (41). Its strong correlation with both the CR/RR dichotomy and OS suggests that *MAP3K15* may serve as a mechanistically relevant marker that complements the surface-derived ImmuScore. Collectively, these findings indicate that RNA-level biomarkers-particularly *MAP3K15*-provide additional explanatory power for the heterogeneous outcomes of *ASXL1*-mutated AML, enabling more refined prognostic stratification when integrated with immunophenotypic and clinical variables.

### Validation of the immuScore in an independent clinical cohort

**Figure 6:**
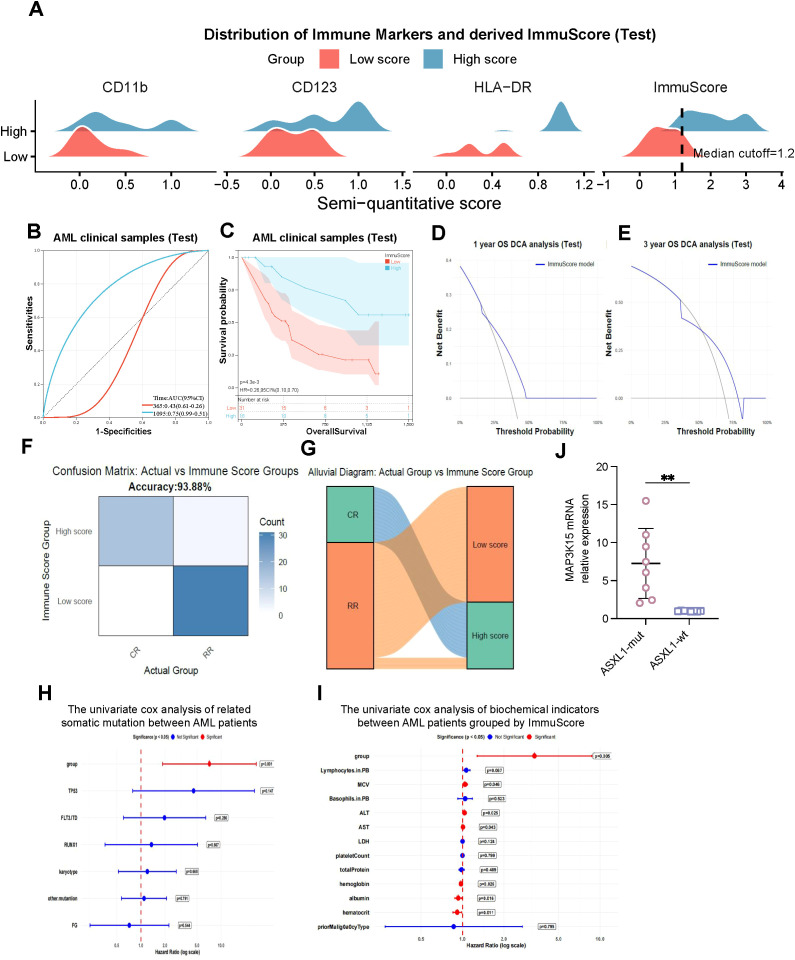
Validation of the ImmuScore model in the independent institutional cohort. **(A)** Distribution of immune marker expression (CD11b, CD123, HLA-DR) stratified by ImmuScore-defined groups in the clinical AML cohort, low-score patients showed reduced CD11b and HLA-DR expression compared with high-score group, while CD123 expression remained low in both groups. **(B)** ROC curve analysis confirming the discriminatory performance of the ImmuScore (AUC = 0.74) in the clinical validation cohort. **(C)** Kaplan-Meier survival curves demonstrating that high ImmuScore patients had significantly longer overall survival compared with low-score patients (*P* < 0.01). **(D, E)** Decision curve analyses (DCA) for 1- and 3-year overall survival, indicating net clinical benefit of the ImmuScore model across relevant thresholds. **(F)** Confusion matrix showing the accuracy (93.88%) of ImmuScore in distinguishing CR from R/R patients. **(G)** Alluvial diagram visualizing the concordance between clinical response (CR vs. R/R) and immune grouping defined by ImmuScore. **(H, I)** Univariate Cox regression analyses of somatic mutations **(H)** and biochemical indicators **(I)** associated with prognosis in AML patients grouped by ImmuScore. **(J)** qPCR validation demonstrating that *MAP3K15* expression was significantly increased in *ASXL1*-mutated patients compared with wild-type controls (*P* < 0.01). ** Representing p <0.01.

To determine whether the ImmuScore developed from the BeatAML cohort could be generalized beyond the discovery dataset, we evaluated its performance in an independent in-house AML cohort (**Table 1**). Consistent with our discovery findings, the validation cohort exhibited clear immunophenotypic separation: patients in the High-Score group displayed markedly distinct expression patterns of CD11b and HLA-DR compared to the Low-Score group (**Figure 6A**). Time-dependent ROC analysis confirmed that the three-marker model maintained strong predictive accuracy, achieving an Area Under the Curve (AUC) of 0.75 for overall survival (OS) (**Figure 6B**). Kaplan-Meier analysis similarly demonstrated that High-Score patients had significantly superior survival (*P* = 0.02; **Figure 6C**). Furthermore, Decision Curve Analysis (DCA) demonstrated a consistent clinical net benefit for the ImmuScore in predicting both 1-year and 3-year OS (**Figures 6D, E**).

**Table 1:** Sample clinical characteristics.

Characteristics	Mean (range)	No. of cases
Sex		
Male		24
Female		25
Age	55.9(18-87)	
<60 years		24
≥60 years		25
WBC		
Mean,×109/L	14.55(0.49-116.84)	
Hemoglobin		
Mean,g/L	74.24(38-119)	
Platelets		
Mean,×109/L	92(2-577)	
BM blasts		
median,%	47.8(20.02-89.1)	
FAB subtype		
M0		0
M1		1
M2		17
M3		0
M4		6
M5		16
M6		0
M7		0
unclassified		9
Karyotype		
Normal karyotype		32
Favorable		2
Other intermediate		9
Adverse		6
Gene mutations		
ASXL1		49
FLT3-ITD/TKD		4
NPM1		1
DNMT3A		2
IDH1/2		9
SRSF2		6
U2AF1		1
RUNX1		4
Immune score		
High risk		31
Low risk		18
Status		
0		19
1		30
Survival time		
<3 years		41
≥3 years		8

We next assessed the alignment between the ImmuScore and actual treatment response. The model distinguished complete remission (CR) from relapsed/refractory (R/R) patients with a high accuracy of 93.88% (**Figure 6F**). An alluvial diagram confirmed substantial concordance between clinical outcomes and ImmuScore assignment (**Figure 6G**), indicating that the immunophenotype-based stratification captures biologically meaningful and clinically relevant differences. This underscores the utility of the ImmuScore as a robust tool for refining risk stratification specifically within the heterogeneous *ASXL1*-mutated population.

To contextualize the observed clinical heterogeneity, univariate Cox analyses identified several molecular and biochemical features associated with prognosis, including *TP53*, *FLT3-ITD*, and *RUNX1* mutations, alongside cytogenetic abnormalities, LDH levels, and inflammatory markers (**Figures 6H, I**). Furthermore, RT-qPCR validation confirmed that *MAP3K15* expression was significantly upregulated in *ASXL1*-mutated patients compared to wild-type cases (*P* < 0.01; **Figure 6J**), reinforcing our transcriptomic findings from the BeatAML dataset. Notably, within the *ASXL1*-mutated cohort, *MAP3K15* expression levels significantly differed between clinical response subgroups; specifically, expression was markedly higher in the R/R group compared to the CR group (**Supplementary Figure 5C**). This further identifies *MAP3K15* as a biologically significant prognostic modifier linked to therapeutic resistance.

It is worth noting that both the BeatAML dataset and our in-house cohort each contained approximately 50 *ASXL1*-mutated cases-representing some of the largest available sample sets for this specific mutation. While power analysis suggested that larger cohorts (n > 244; **Supplementary Figure 2C**) would be ideal for maximum statistical power, extensive searches across public repositories (e.g., GEO) failed to yield additional datasets with adequate clinical and flow-cytometric annotation. To address potential small-sample bias and ensure the robustness of our statistical inference, we applied Firth penalized regression. This method reduced bias in parameter estimation and improved model stability, thereby enhancing the credibility of our results despite unavoidable sample-size constraints.

Collectively, the validation cohort reproduced the key findings of the discovery phase, demonstrating that the ImmuScore is a robust, biologically grounded, and clinically applicable tool for refining risk stratification in AML-particularly within the challenging *ASXL1*-mutated subgroup.

### ImmuScore predicts sensitivity to chemotherapy and targeted agents in AML

Given that the ImmuScore was robustly associated with survival and clinical response in both the BeatAML and validation cohorts, we next examined whether this immunophenotype-based classifier reflected disparities in therapeutic susceptibility. In both datasets, drug-response measurements were normalized as Z-scores, where higher values correspond to reduced sensitivity. This analysis allowed us to determine whether the ImmuScore effectively captures functional drug-resistance phenotypes.

**Figure 7:**
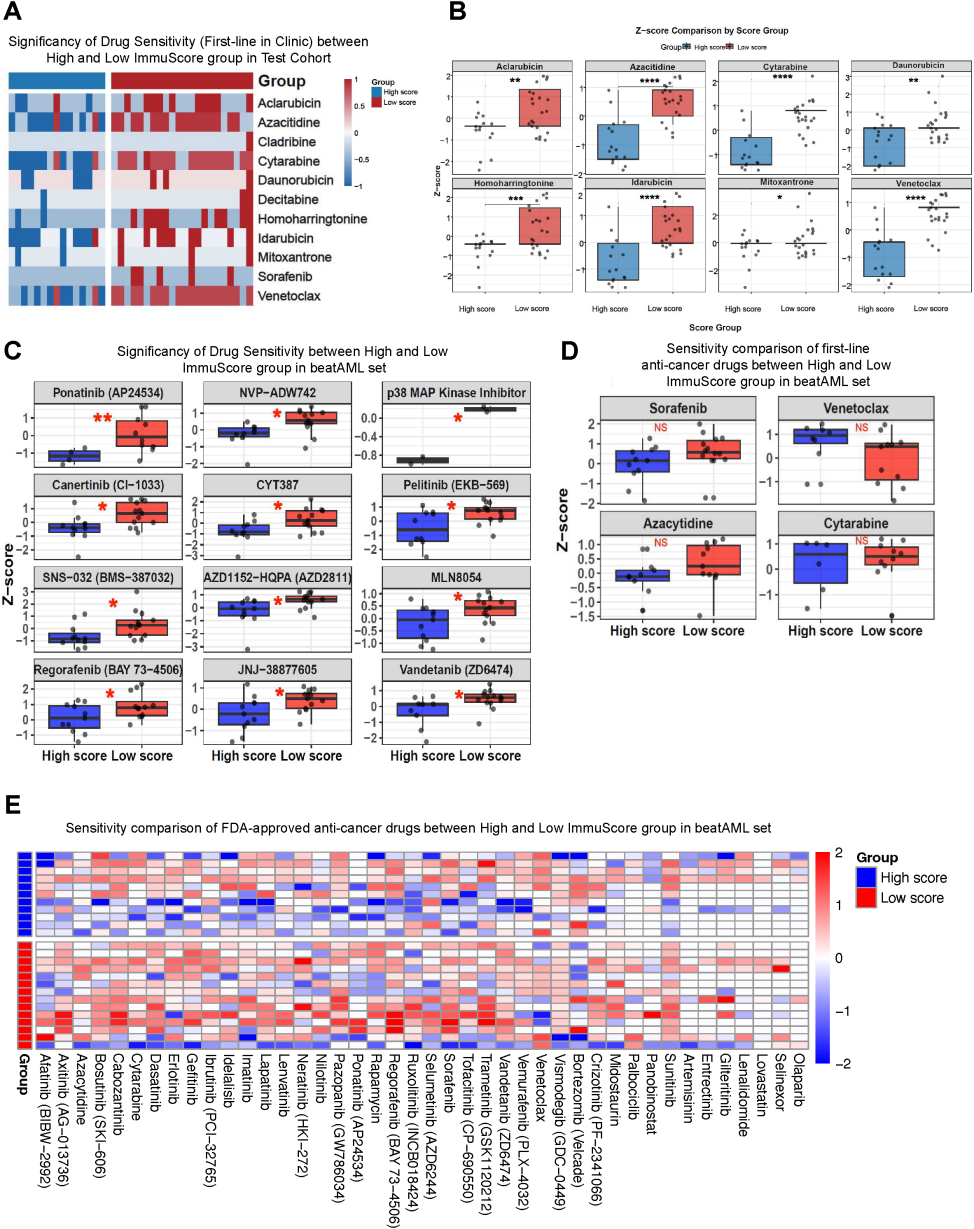
Correlation between ImmuScore and therapeutic resistance. **(A, B)** Analysis of first-line clinical drugs in the in-house AML cohort, showing Z-score-based drug sensitivity differences between high- and low-ImmuScore groups. Patients with low ImmuScore exhibited significantly higher Z-scores, indicating relative resistance to multiple frontline agents, whereas high-score patients showed lower Z-scores and greater drug sensitivity. **(C)** Validation in the BeatAML dataset demonstrated increased sensitivity patterns in high-ImmuScore patients and relative resistance in Low-ImmuScore patients. **(D)** Comparison of first-line anti-leukemic drugs in BeatAML revealed that high-ImmuScore patients showed increased sensitivity to sorafenib and azacitidine, with cytarabine and venetoclax showing a similar but non-significant trend. **(E)** Heatmap summarizing the sensitivity profiles of FDA-approved anti-cancer drugs in BeatAML, confirming widespread resistance in the Low-ImmuScore subgroup. * indicates p <0.05; ** indicates p <0.01; *** indicates p <0.001; **** indicates p <0.0001.

We first assessed drug sensitivity in our institutional cohort using standard frontline chemotherapeutic agents. Patients in the High ImmuScore group consistently exhibited significantly lower Z-scores for aclarubicin, azacitidine, cytarabine, daunorubicin, and idarubicin (**Figures 7A, B**), indicating a more pharmacologically sensitive phenotype. In contrast, patients with Low ImmuScores showed markedly elevated Z-scores, consistent with a reduced response to chemotherapy. These results demonstrate that the immunophenotypic signatures underlying the ImmuScore are tightly linked to functional drug response in real-world clinical samples.

To validate these findings, we extended the analysis to the BeatAML dataset, which provides comprehensive *ex vivo* drug-sensitivity profiles for a large panel of targeted agents. A pattern consistent with our clinical observations emerged: High-Score patients displayed broadly lower Z-scores across multiple small-molecule inhibitors with diverse mechanisms-including ponatinib, NVP-ADW742, pelitinib, MLN8054, regorafenib, and vandetanib (**Figure 7C**). This observation suggests that the Low ImmuScore group exhibits a generalized resistance phenotype rather than vulnerability limited to a specific drug class. When focusing specifically on clinically relevant agents (**Figure 7D**), sorafenib and azacitidine demonstrated significantly higher sensitivity in the High-Score group. While cytarabine and venetoclax did not reach statistical significance, the overall distribution trended toward increased susceptibility among High-Score patients. A broader assessment of FDA-approved anti-cancer drugs (**Figure 7E**) further reinforced this trend, as High-Score patients exhibited a global shift toward lower Z-scores across the full drug panel.

Collectively, these findings indicate that the ImmuScore is not only a prognostic classifier but also a functional marker of therapeutic vulnerability in AML. Patients with High ImmuScores consistently exhibit lower drug-resistance signatures across independent datasets, whereas Low-Score patients maintain a relatively resistant profile. This cross-cohort reproducibility underscores the biological validity of the ImmuScore and highlights its potential utility in guiding individualized therapeutic decisions-specifically by identifying Low-Score patients who are at high risk of treatment failure and may require treatment intensification or alternative therapeutic strategies.

## Discussion

*ASXL1* is one of the most frequently mutated epigenetic regulators in AML and is formally categorized as an adverse-risk lesion within the ELN-2022 classification framework (42, 43). Consistent with previous reports, our multivariable analysis in the BeatAML cohort confirmed *ASXL1* mutation as an independent poor prognostic factor. However, when *ASXL1*-mutated patients were compared with those lacking any other adverse genetic lesions, overall survival did not differ significantly (44, 45). This key observation indicates that the prognostic effect of *ASXL1* is not monolithic, and that its clinical impact is strongly influenced by biological context. Similar to how the *FLT3-ITD* allelic ratio modifies the effect of *NPM1* mutation, our findings suggest that *ASXL1*-mutated AML requires additional “modifier factors” to fully determine clinical behavior (46).

To decipher this heterogeneity, we compared *ASXL1*-mutated patients with divergent therapeutic outcomes-CR versus R/R. This comparison revealed that despite sharing the same defining mutation, these patients displayed striking differences in drug response, immunophenotype, and survival (47). Among multiple layers of data examined-including clinical parameters, comutation patterns, RNA-Seq expression, and ex vivo drug response-immunophenotypic differences emerged as the most robust, consistent, and clinically actionable discriminator. Five surface antigens differed significantly between CR and R/R patients, and three of them-CD11b, CD123, and HLA-DR-are routinely included in diagnostic AML flow cytometry (48, 49). Their biological functions further support their relevance: CD11b reflects myeloid maturation, CD123 is associated with leukemic stemness and therapy resistance, and HLA-DR is critical for antigen presentation and immune engagement.

Based on these three clinically accessible markers, we developed a practical immunophenotypic classifier, the ImmuScore. The score effectively stratified patients into two biologically and clinically distinct subgroups in both the BeatAML discovery cohort and our independent institutional cohort. Notably, the ImmuScore demonstrated prognostic accuracy comparable to the ELN-2017 system, while providing a more refined stratification within the *ASXL1*-mutated subgroup. Beyond survival, the ImmuScore correlated with biochemical abnormalities, cytogenetic complexity, and known high-risk mutations, indicating that it captures broader aspects of disease biology (50).

Crucially, the ImmuScore also predicted therapeutic vulnerability. Low-score patients exhibited markedly reduced sensitivity to both chemotherapeutic agents and multiple targeted inhibitors across two independent datasets (BeatAML and our in-house cohort). This functional association strengthens the biological relevance of the score and provides a mechanistic explanation for why certain *ASXL1*-mutated patients fail frontline therapy despite sharing the same driver alteration. Such dual relevance-prognostic and predictive-highlights the translational value of immunophenotypic profiling in guiding treatment strategies.

Beyond immunophenotype, transcriptomic profiling identified *MAP3K15* as an additional molecular marker that complements the ImmuScore. *MAP3K15* was consistently upregulated in *ASXL1*-mutated patients and significantly associated with survival. Given its known role in stress-response signaling pathways, *MAP3K15* may represent a mechanistically meaningful contributor to treatment resistance and warrants further functional investigation.

Several limitations should be acknowledged. In addition, we acknowledge that the number of ASXL1-mutated patients included in the present study remains relatively limited. Therefore, the current ImmuScore should be interpreted as a preliminary and exploratory scoring framework rather than a fully optimized predictive model. Although our ASXL1-mutated sample size (~50 patients per dataset) represents one of the largest currently available, statistical power remains limited, as confirmed by power analysis. Extensive searches of GEO and other repositories did not identify additional suitable datasets. To mitigate small-sample bias, we used Firth penalized regression, which is well suited for sparse-event data and enhances reliability under these constraints. Second, our immunophenotypic scoring was derived from a single-center flow cytometry protocol; thus, inter-laboratory standardization will be essential for future clinical adoption. Third, although we incorporated multiple biological layers, residual confounding from co-mutations and treatment heterogeneity cannot be fully excluded.

In summary, our study demonstrates that *ASXL1*-mutated AML is not a biologically homogeneous high-risk entity, but rather a genetically defined group with substantial internal heterogeneity. By integrating surface immunophenotyping with genomic and transcriptomic features, we developed the ImmuScore, a simple and clinically practical modifier that enables more refined risk stratification than mutation status alone. The score’s ability to predict both survival and therapy responsiveness highlights its translational potential. Prospective multicenter validation, standardization of antigen scoring, and incorporation of ImmuScore into treatment decision algorithms and MRD monitoring will be important next steps. Collectively, these findings support a shift toward genotype-phenotype integrative classification, enabling more precise identification of high-risk *ASXL1*-mutated AML and informing personalized therapeutic strategies.

## Data Availability

The original contributions presented in the study are included in the article/**Supplementary Material**. Further inquiries can be directed to the corresponding authors.

## Supplementary material

Supplementary Figure 1 Univariate analysis of clinical and genetic prognosticators. Forest plot summarizing hazard ratios (HRs) and 95% confidence intervals for multiple variables, including demographic factors (e.g., age, sex), prior malignancy history, treatment exposures, peripheral blood cell composition, biochemical parameters (e.g., LDH, ALT, AST, creatinine, albumin), and genetic alterations (e.g., *FLT3-ITD*, *NPM1*, *RUNX1*, *ASXL1*, *TP53*, *CEBPA*, cytogenetic abnormalities). Red dots represent variables with non-significant associations, and blue dots represent significant predictors of overall survival. Variables with HR > 1 are associated with increased risk of adverse outcomes, while HR < 1 indicates protective effects.

Supplementary Figure 2 Model comparison, power analysis, and immune deconvolution. **(A, B)** ROC curve analysis comparing the predictive performance of the 3-marker and 5-marker models for overall survival at 1 year [365 days, **(A)**] and 2 years [730 days, **(B)**]. The 3-marker model demonstrated higher AUC values compared to the 5-marker model at both time points, indicating superior discriminatory capacity. **(C)** Sample size estimation for the 3-marker model. A minimum of 244 patients was predicted to be required to achieve robust statistical power, highlighting the limitations of small sample sizes in the current datasets. **(D)** Immune cell composition differences between CR (complete response) and RR (refractory/relapsed) groups, as inferred by CIBERSORT analysis. While most immune subsets showed no significant differences, regulatory T cells (Tregs) were significantly enriched in the CR group compared to RR patients (*P* < 0.001).

Supplementary Figure 3 Schematic overview of the study workflow.

Supplementary Figure 4 Performance of the 5-marker ImmuScore model in AML subgroups. **(A–E)** Prognostic evaluation of the 5-marker ImmuScore in *ASXL1*-mutated patients. **(F, G)**. Classification accuracy in *ASXL1*-mutated patients.

Supplementary Figure 5 Validation and Specificity Assessment of the 3-antigen ImmuScore in ASXL1 Wild-type AML. **(A, B)**. Specificity validation in the *ASXL1*-wildtype (*ASXL1*^WT^) cohort. Confusion matrix **(A)** and alluvial diagram **(B)** showing a marked reduction in classification accuracy (53.22%) when the model is applied to patients lacking *ASXL1* mutations. **(C)**. Differential expression analysis of *MAP3K15* in the *ASXL1*-mutated cohort.
