# Supplementary figures and images for: Prognostic heterogeneity in ASXL1-mutated AML and refinement by an immunophenotype-based score
Source: Front Oncol. 2026 May 19;16:1780304. doi: 10.3389/fonc.2026.1780304 (PMC13225974; doi:10.3389/fonc.2026.1780304)

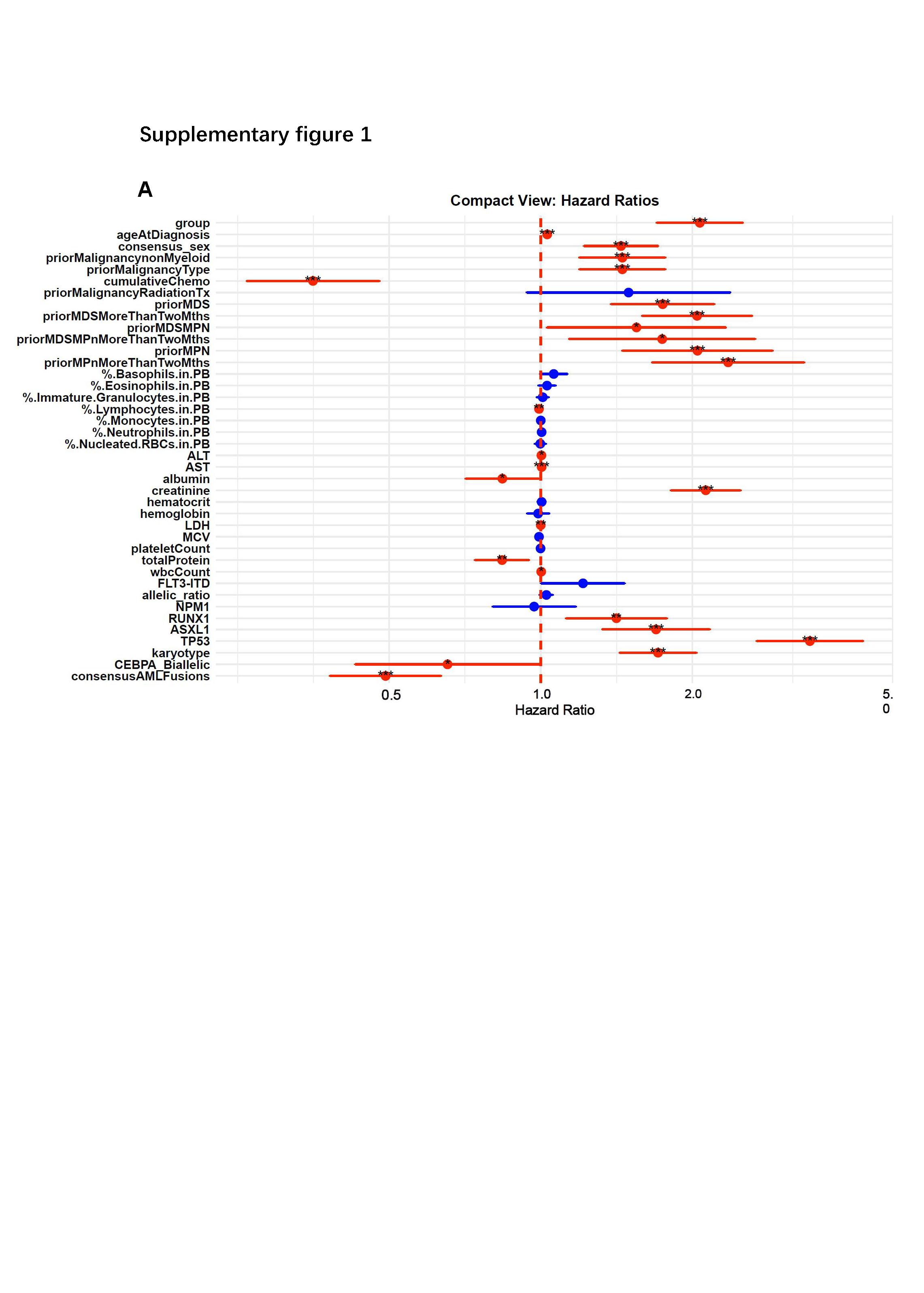

Supplement: Supplementary Figure 1 — Univariate analysis of clinical and genetic prognosticators. Forest plot summarizing hazard ratios (HRs) and 95% confidence intervals for multiple variables, including demographic factors (e.g., age, sex), prior malignancy history, treatment exposures, peripheral blood cell composition, biochemical parameters (e.g., LDH, ALT, AST, creatinine, albumin), and genetic alterations (e.g., FLT3-ITD, NPM1, RUNX1, ASXL1, TP53, CEBPA, cytogenetic abnormalities). Red dots represent variables with non-significant associations, and blue dots represent significant predictors of overall survival. Variables with HR > 1 are associated with increased risk of adverse outcomes, while HR < 1 indicates protective effects. [file Image1.tif]

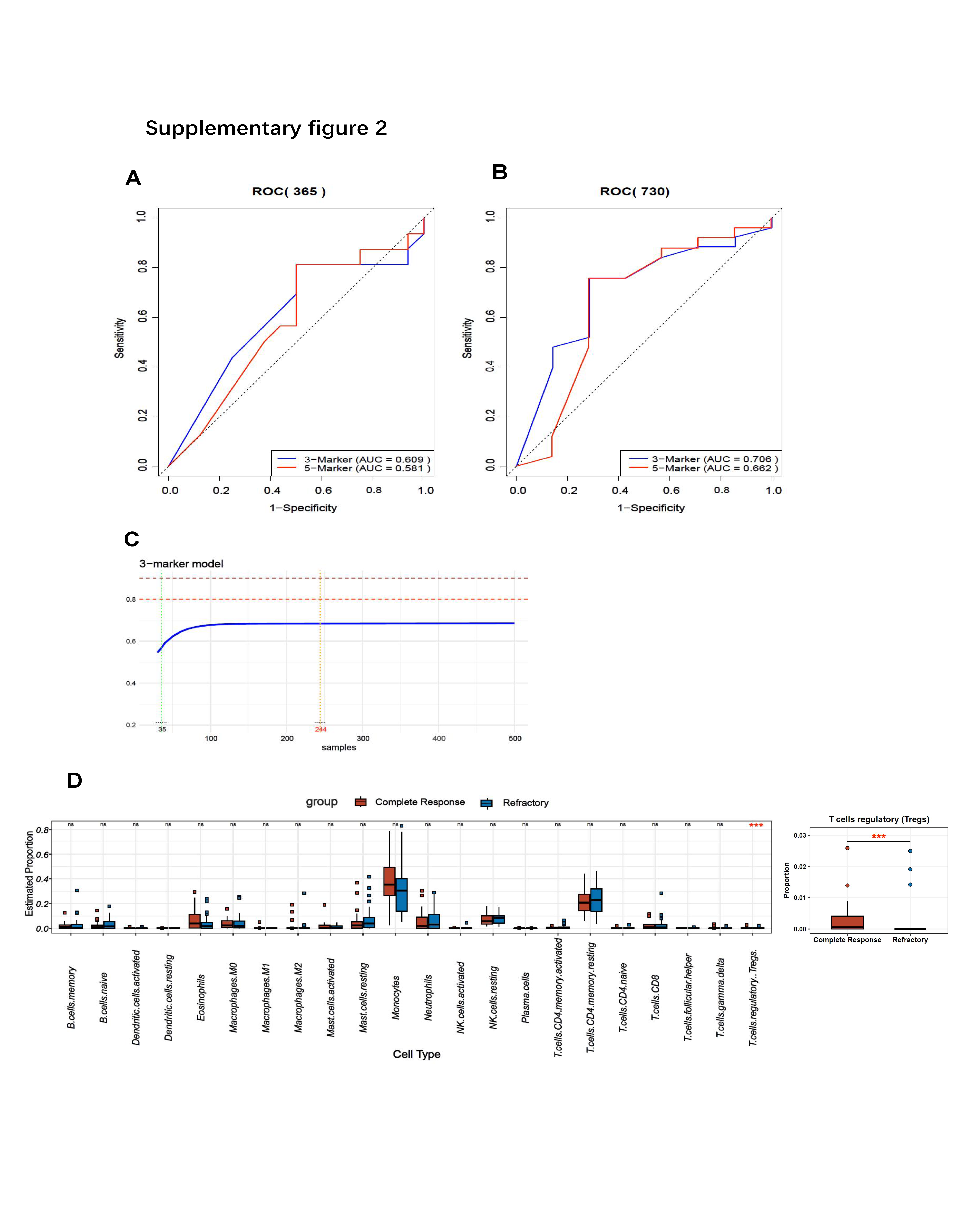

Supplement: Supplementary Figure 2 — Model comparison, power analysis, and immune deconvolution. (A, B) ROC curve analysis comparing the predictive performance of the 3-marker and 5-marker models for overall survival at 1 year [365 days, (A)] and 2 years [730 days, (B)]. The 3-marker model demonstrated higher AUC values compared to the 5-marker model at both time points, indicating superior discriminatory capacity. (C) Sample size estimation for the 3-marker model. A minimum of 244 patients was predicted to be required to achieve robust statistical power, highlighting the limitations of small sample sizes in the current datasets. (D) Immune cell composition differences between CR (complete response) and RR (refractory/relapsed) groups, as inferred by CIBERSORT analysis. While most immune subsets showed no significant differences, regulatory T cells (Tregs) were significantly enriched in the CR group compared to RR patients (P < 0.001). [file Image2.tif]

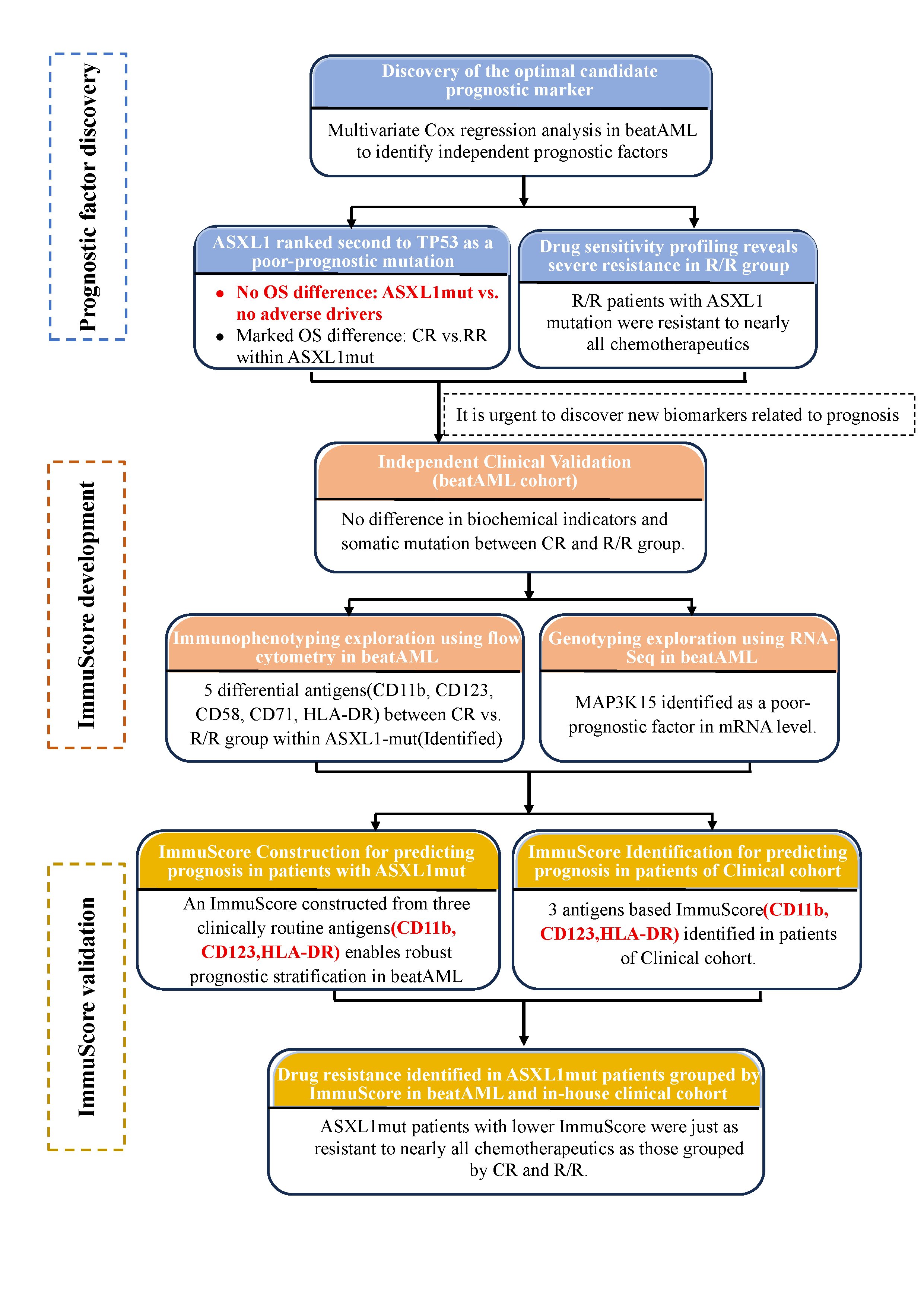

Supplement: Supplementary Figure 3 — Schematic overview of the study workflow. [file Image3.tif]

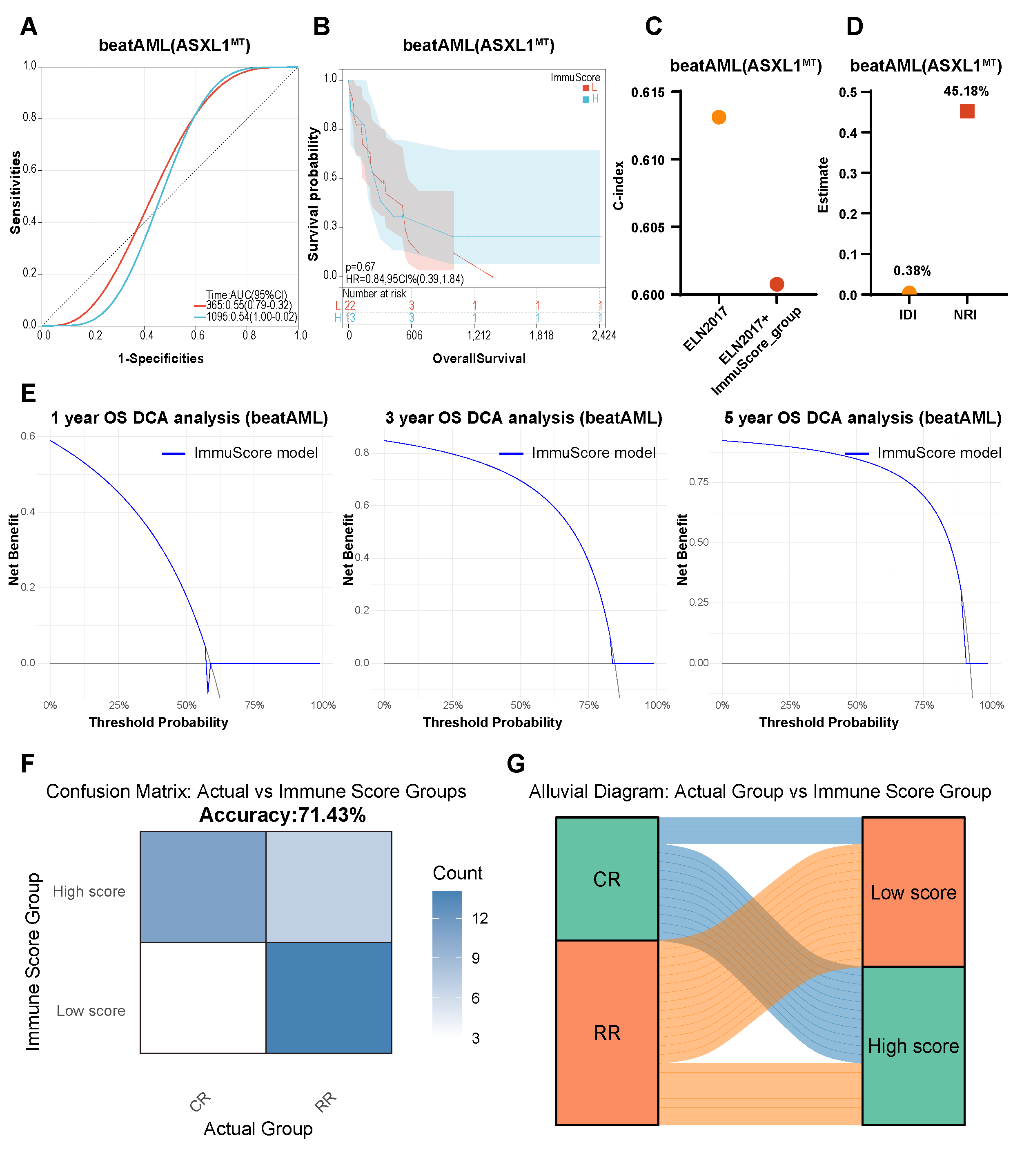

Supplement: Supplementary Figure 4 — Performance of the 5-marker ImmuScore model in AML subgroups. (A–E) Prognostic evaluation of the 5-marker ImmuScore in ASXL1-mutated patients. (F, G). Classification accuracy in ASXL1-mutated patients. [file Image4.tif]

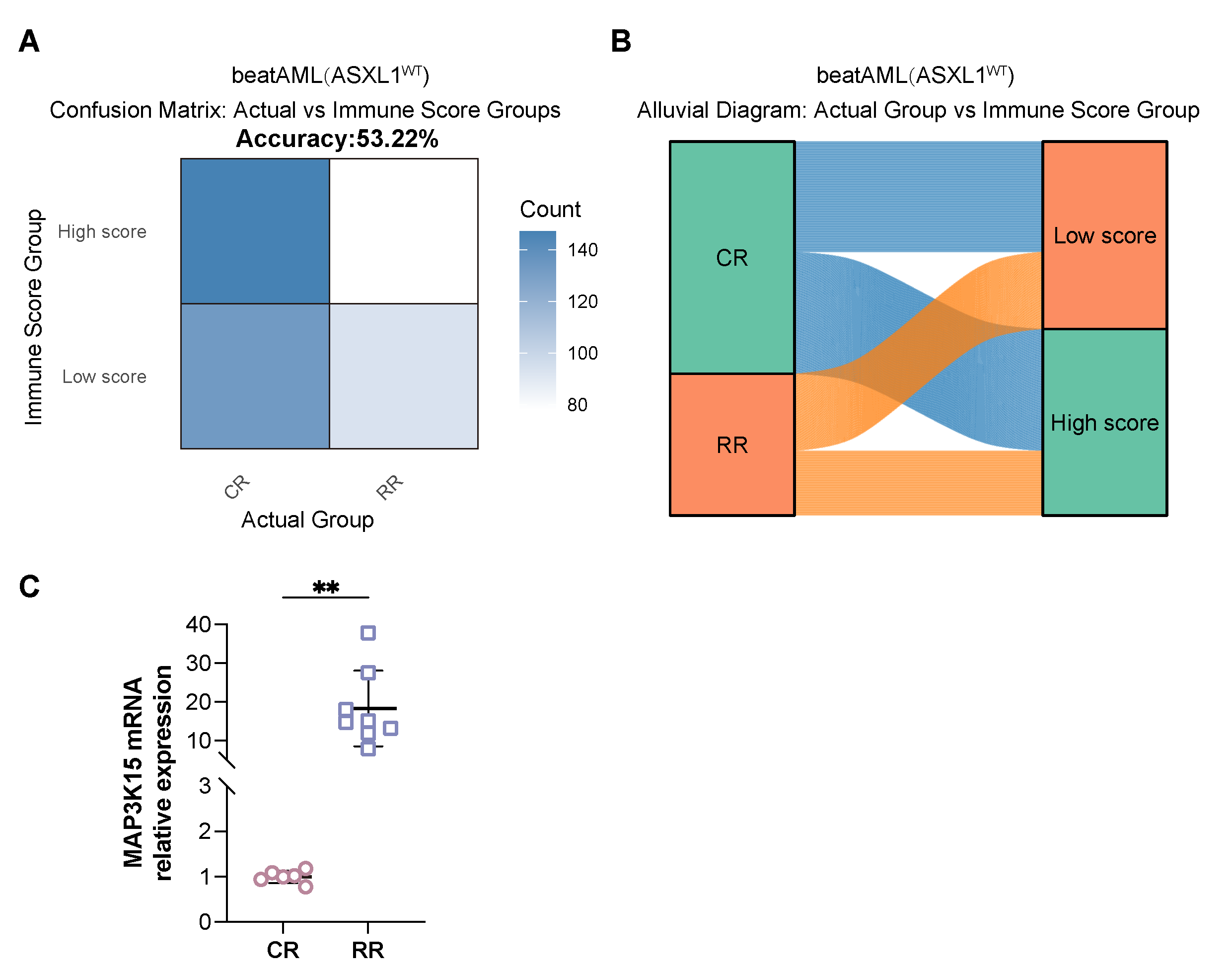

Supplement: Supplementary Figure 5 — Validation and Specificity Assessment of the 3-antigen ImmuScore in ASXL1 Wild-type AML. (A, B). Specificity validation in the ASXL1-wildtype (ASXL1WT) cohort. Confusion matrix (A) and alluvial diagram (B) showing a marked reduction in classification accuracy (53.22%) when the model is applied to patients lacking ASXL1 mutations. (C). Differential expression analysis of MAP3K15 in the ASXL1-mutated cohort. [file Image5.tif]
